# Genomics of rare genetic diseases—experiences from India

**DOI:** 10.1186/s40246-019-0215-5

**Published:** 2019-09-25

**Authors:** The GUaRDIAN Consortium, Anjali Bajaj, Samatha Mathew, Shamsudheen Karuthedath Vellarikkal, Ambily Sivadas, Rahul C. Bhoyar, Kandarp Joshi, Abhinav Jain, Anushree Mishra, Ankit Verma, Rijith Jayarajan, A. Nalini, A. Ravi Kumar, A. T. Arasar Seeralar, Aayush Gupta, Achal K. Srivastava, Aditi Joshi, Aditi Sinha, Aditya Jandial, Afreen Khan, Akhilesh K. Sonakar, Alex Chandy, Aman Sharma, Ambuj Roy, Amit Rawat, Amitabh Biswas, Andrew Vanlalawma, Anita Chaudhary, Anita Chopra, Ankit Panday, Ankit Sabharwal, Ankita Mitra, Ankita Narang, Anna Rajab, Anoop Kumar, Anoop Singh Gurjar, Anop Singh Ranawat, R. I. Anu, Anup Kumar Tiwary, Anuradha, Aquil Kalanad, Aradhana Mathur, Arjun Lakshman, Arushi Batra, Arvind Bagga, Ashish Aggarwal, Ashok Gupta, Ashu Rastogi, P. K. Aslam, V. Astha, Aswin Nair, E. P. Athulya, Atri Chatterjee, Atul Jindal, Atul Kumar Kashyap, B. Priyadarshini, Babu Ram Thapa, Balram Bhargava, Balram Sharma, Bani Jolly, Bharath Ram Uppilli, Bharathi Balachander, Bhim Shankar, Bibhas Kar, B. K. Binukumar, C. Lalchhandama, Chaitanya Datar, Chetana Sachidanandan, D. C. Master, Daisy Khera, Debashish Chowdhury, Debashish Danda, Deepak Kumar, Deepika Pandhi, Deepti Siddharthan, Disha Sharma, Divya Pachat, Brijesh Sharma, Durga Rao Vegulada, G. S. R. S. N. K. Naidu, G. Padma, G. Vishnu Priya, Gautam Sharma, R. Gauthamen, Geeta Govindaraj, George M. Varghese, S. Gireesh, Gopi Krishnan Unnikrishnan, S. A. Hafiz, K. R. Hazeena, Heena Dhiman, Hema Singh, Hrishikesh Sarkar, Istaq Ahmed, Jagadeesh Menon, Jatinder Goraya, Jennifer Mathew, Jineesh Thottath, Jitendra K. Sahu, Jitendra Oswal, John Menachery, Judith Mary Hariprakash, K. Bhargava, K. K. Talwar, K. M. Cherian, K. P. Aravindan, K. Pramila, K. Saroja, K. Shantaraman, Kavita Pandhare, Kiran Kumar Mandapati, P. Kiran, Kotha Rakesh, Krati Shah, C. Krishnan, Kriti Shah, Kuldeep Singh, Kuljeet Anand, Lalawmpuii Pachuau, Laxmisha Chandrashekar, Liza Rajasekhar, Lopamudra Mishra, M. V. Padma, Madhulika Kabra, Madhumita Roy Chowdhary, Malika Seth, Maneesh Rai, Manish Kumar, Manish Parakh, Manisha Goyal, Manisha Gurjar, Manisha Sahay, Mercy Rophina, Mitali Mukerji, Mohammed Ali, Mohammed Faruq, Mohandas Nair Karippoth, Mohit Kumar Divakar, M. P. Jayakrishnan, Mukesh Kumar, Mukta Poojary, Mukund A. Prabhu, Nachimuthu Senthil Kumar, Nadeem Rais, Nalini Bhaskaranand, Narendra Kumar Bagri, Naveen Sankhyan, Neeraj Awasthy, Neeraj Gupta, Neeraj Parakh, Neerja Gupta, Neetu Bhari, Neetu Kushwaha, Neha Sharma, Neha Virmani, Nilanjan Kundu, Nishad Plakkal, Nishu Tyagi, Nita Radhakrishnan, Nitish Naik, Nitish Rai, Nivedita Mondal, Nupur Bhargava, Pankaj Hari, Paras Sehgal, Piyush Kumar, Pooja Chauhan, Pooja Mailankody, Pooja Sharma, Poonam Parakh, Pragya A. Nair, Praloy Chakraborty, Prasanna Kumar Shirol, Pratibha Singh, Pratosh Gangadhar, Prawin Kumar, Purna Chandra, R. Krishnan, R. Srilakshmi, R. Sriranga Lakshmi, R. Anantharaman, Radha Mahadevan, Rahul Mahajan, Rajasubramaniam Shanmugam, Rajat Sharma, V. R. Rajendran, Rajinder K. Dhamija, Rajit Pillai Ramanan, Rajive Kumar, A. R. Rajneesh, Rajnish Juneja, Rakesh Aggarwal, Rakesh Sahay, S. Ramakrishnan, Ranjith Narayanan, Ravindra Shukla, Remya Koshy, Renu Kumari, Richa Chaudhary, Richa Jain, Riyaz Arakkal, Roopa Rajan, Rowmika Ravi, S. Baruah, S. Sitaraman, Sadandandavalli Retnaswami Chandra, Saia Chenkual, V. Sailaja, Sakshi Ambawat, Samhita Panda, Sana Zahra, Sanchit Kumar, Sandeep Arora, Sandeep Mathur, Sandeep Seth, P. Sandhya, Sangam Goswami, Sangita Paul, Sanjay Pandey, Santharaman Kalyanaraman, Saroj Patnaik, Saruchi Wadhwa, Sathi Venu, Satyan Nanda, Saumya Panda, Saurabh Chopra, Saurabh Singh, P. Savinitha, Seema Kapoor, Sesh Sivadasan, G. Sethuraman, Shaista Parveen Khan, C. V. Shaji, Shanmugam Gurusamy, Sheffali Gulati, Shrey Gandhi, Sivaprakash Ramalingam, Smita Nath, Somesh Kumar, Sona Sathian, Sonal Lakhani, Soumya S. Nair, Soumya Sundaram, Sourav Ghosh, Sree Bhushan Raju, Sreejith Valappil, Sreelata Nair, Srikanth Kadyada Puttaiah, Sruthi S. Nair, Suja K. Geevarghese, Sujata Mohanty, Sujay Khandpur, Suman Jain, Sumeet, Sumit Sharma, Suruchi Trehan, Suvasini Sharma, Sweta jain, Swetha Jain, Tarun Kumar Badam, S. Umamaheswari, Utkarsh Gaharwar, Uzma Shamim, Vadlamudi Raghavendra Rao, Vamsi Krishna, Vandana Jain, Varun Suroliya, Varuna Vyas, Veena Vedartham, S. Venketesh, Vigneshwar Senthivel, Vijaykumar Bhavi, Vilas Jadhav, Vinay Gera, Vishal Dixit, Vishal Gupta, Vishnu Agarwal, V. Y. Vishnu, Vishu Gupta, K. V. Vysakha, Yugal K. Sharma, Samir K. Brahmachari, Vinod Scaria, Sridhar Sivasubbu, Sridhar Sivasubbu, Vinod Scaria

**Affiliations:** 1 CSIR Institute of Genomics and Integrative Biology, Delhi, 110025 India; 2 Academy of Scientific and Innovative Research, Delhi, India; 3 National Institute of Mental Health and Neuro Sciences, Bengaluru, India; 4 Thalassemia and Sickle Cell Society, Hyderabad, India; 5 Institute of Child Health and Hospital for Children, Chennai, India; 6 Dr. DY Patil Medical College and hospital, Pune, India; 7 All India Institute of Medical Sciences, Delhi, India; 8 Post Graduate Institute of Medical Education and Research, Chandigarh, India; 9 Govt Medical college Kozhikode, Kerala, India; 10 Galgotias University, Greater Noida, India; 11 Mizoram University, Aizawl, India; 12 Sawai Man Singh Medical College, Jaipur, India; 13 Wadia Children Hospital, Mumbai, India; 14 VPS Healthcare, Abu Dhabi, United Arab Emirates; 15 Government Medical College, Barmer, India; 16 MVR Cancer Centre and Research Institute, Calicut, India; 17 Government Medical College, Haldwani, India; 18 Government Medical College Kozhikode, Kozhikode, India; 19 JK Lone Hospital, Sawai Man Singh Medical College, Jaipur, India; 20 Christian Medical College Hospital, Vellore, India; 21 Vardhman Mahavir Medical College & Safdurjung Hospital, Delhi, India; 22 All India Institute of Medical Sciences, Raipur, India; 23 St. Johns Medical College Hospital, Bangalore, India; 24 Lakeshore Hospital, Kochi, India; 25 Madras Medical Mission, Chennai, India; 26 Department of Pathology, Civil Hospital Aizawl, Aizawl, India; 27 Sahyadri Medical Genetics and Tissue Engineering Facility, Pune, India; 28 Medical College, Baroda, India; 29 All India Institute of Medical Sciences, Jodhpur, India; 30 GB Pant Hospital, Delhi, India; 31 University College of Medical Sciences and Guru Teg Bahadur Hospital, Delhi, India; 32 Malabar Institute of Medical Sciences, Calicut, India; 33 Dr. Ram Manohar Lohia hospital, Delhi, India; 34 Genes N life Healthcare, Hyderabad, India; 35 Christian Medical College, Vellore, India; 36 sree Chithra Tirunal Institute of Medical Sciences and Technology, Trivandrum, India; 37 General Hospital, Thiruvananthapuram, India; 38 Noorul Islam Multi Speciality Hospital, Thiruvananthapuram, India; 39 Sawai Man Singh Hospital, Jaipur, India; 40 Holy Family Hospital, Mumbai, India; 41 Dayanand Medical College & Hospital, Ludhiana, India; 42 Calicut Medical College, Calicut, India; 43 Government Medical College Kozhikode, Calicut, India; 44 Bharati Vidyapeeth Medical College and Hospital, Pune, India; 45 Rajagiri Hospital, Aluva, India; 46 Max Super Speciality Hospital, Delhi, India; 47 Frontier Lifeline and Dr. K. M. Cherian Heart Foundation, Chennai, India; 48 Tirunelveli Medical College, Tirunelveli, India; 49 Malabar Cancer Centre, Kannur, India; 50 Osmania Medical College, Hyderabad, India; 51 ONE Centre for Rheumatology & Genetics, Vadodara, India; 52 Institute of Maternal & Child Health, Government Medical College, Kozhikode, India; 53 Isha Hospital, Vadodara, India; 54 Civil Hospital Aizawl, Aizawl, India; 55 Jawaharlal Institute of Postgraduate Medical Education and Research, Puducherry, India; 56 Nizam’s Institute of Medical Sciences, Hyderabad, India; 57 Kasturba Medical College, Mangalore, India; 58 Dr. Sampurnanand Medical College, Jodhpur, India; 59 J K Lone Hospital, Sawai Man Singh Hospital, Jaipur, India; 60 Amritha institute of medical sciences, Cochin, India; 61 Chowpatty Medical Center, Mumbai, India; 62 Hitech Medicare Hospital, Udupi, India; 63 Max Hospital, Delhi, India; 64 Lady Hardinge Medical College, Delhi, India; 65 Sakhiya Skin Clinic, Surat, India; 66 Dr. Nilanjan Kundu’s Nursing Home, Bankura, India; 67 Jawaharlal Institute of Postgraduate Medical Education & Research, Puducherry, India; 68 Super Speciality Pediatric Hospital and PG Teaching Institute, Noida, India; 69 Katihar Medical College, Dalan, India; 70 National Institute of Mental Health and Neurosciences, Bengaluru, India; 71 Pramukhswami Medical College, Karamsad, India; 72 Safdarjung Hospital, Delhi, India; 73 Organization for Rare Diseases, Jodhpur, India; 74 PVS Hospital, Calicut, India; 75 MES Medical College, Malappuram, India; 76 The Tamil Nadu Dr. M.G.R. Medical University, Chennai, India; 77 Frontier Lifeline Hospital, Chennai, India; 78 ICMR-National Institute of Research in Tribal Health, Jabalpur, India; 79 Base Hospital Delhi Cantt, Delhi, India; 80 Aster Malabar Institute of Medical Sciences Hospital, Kozhikode, India; 81 Osmania General Hospital, Hyderabad, India; 82 KMCT Medical College Hospital, Kozhikode, India; 83 Kannur Medical College, Kannur, India; 84 Tezpur University, Tezpur, India; 85 Maulana Azad Medical College, Delhi, India; 86 Command Hospital Air Force, Bengaluru, India; 87 Govind Ballabh Pant Institute of Postgraduate medical education and research, Delhi, India; 88 Army Research and Referral Hospital, Delhi, India; 89 KPC Medical College, Kolkata, India; 90 BLK Super Specialty Hospital, Delhi, India; 91 Maulana Azad Medical College, Lok Nayak Jai Prakash Narayan Hospital, Delhi, India; 92 TD Medical College, Allappuzha, India; 93 Kannur Dental College, Kannur, India; 94 Lifeline Super Specialty Hospital, Pathanamthitta, India; 95 Mar Baselios Medical Mission Hospital, Ernakulam, India; 96 Lady Harding Medical College, Delhi, India; 97 Devadoss Multi Speciality Hospital, Madurai, India; 98 Pudhuvai Life Line Hospital, Puducherry, India; 99 Osmania University, Hyderabad, India; 100 Sri Satya Sai Institute of Higher Learning, Puttaparthy, India; 101 Aarya Child Epilepsy and Neurology Clinic, Kolhapur, India; 102 Base Hospital, Lucknow, India; 103 Sreechithra Tirunal Institute of Medical Sciences and Technology, Trivandrum, India

**Keywords:** Rare disease, Genomics, India, Genetic diversity, Diagnostics, GUaRDIAN, Zebrafish, IPSCs, Patient support

## Abstract

Home to a culturally heterogeneous population, India is also a melting pot of genetic diversity. The population architecture characterized by multiple endogamous groups with specific marriage patterns, including the widely prevalent practice of consanguinity, not only makes the Indian population distinct from rest of the world but also provides a unique advantage and niche to understand genetic diseases. Centuries of genetic isolation of population groups have amplified the founder effects, contributing to high prevalence of recessive alleles, which translates into genetic diseases, including rare genetic diseases in India.

Rare genetic diseases are becoming a public health concern in India because a large population size of close to a billion people would essentially translate to a huge disease burden for even the rarest of the rare diseases. Genomics-based approaches have been demonstrated to accelerate the diagnosis of rare genetic diseases and reduce the socio-economic burden. The Genomics for Understanding Rare Diseases: India Alliance Network (GUaRDIAN) stands for providing genomic solutions for rare diseases in India. The consortium aims to establish a unique collaborative framework in health care planning, implementation, and delivery in the specific area of rare genetic diseases. It is a nation-wide collaborative research initiative catering to rare diseases across multiple cohorts, with over 240 clinician/scientist collaborators across 70 major medical/research centers. Within the GUaRDIAN framework, clinicians refer rare disease patients, generate whole genome or exome datasets followed by computational analysis of the data for identifying the causal pathogenic variations. The outcomes of GUaRDIAN are being translated as community services through a suitable platform providing low-cost diagnostic assays in India. In addition to GUaRDIAN, several genomic investigations for diseased and healthy population are being undertaken in the country to solve the rare disease dilemma.

In summary, rare diseases contribute to a significant disease burden in India. Genomics-based solutions can enable accelerated diagnosis and management of rare diseases. We discuss how a collaborative research initiative such as GUaRDIAN can provide a nation-wide framework to cater to the rare disease community of India.

## Background

### Population architecture and genetic diversity in India

India is the sixth largest country in the world in terms of its geographical area and the second largest country in population density. The people of the country are diverse in terms of their social, linguistic, cultural, and racial backgrounds. Evolutionarily, the Indian subcontinent has been a corridor for different migratory waves arising from Africa, through land as well as coastline routes [1, 2]. Genetic studies have shown that there are four distinct ancestral groups in mainland India, and a separate ancestry in the Andaman and Nicobar islands [3, 4]. On the basis of ethno-racial grounds, the four major groups in India can be classified as the Caucasoids, Australoids, Mongoloids, and Negritos. The Indian population comprises of over 4000 anthropologically distinct groups speaking more than 300 languages [5], suggesting that linguistic stratification is highly tied to the geographical niches of each sub-population [6–10]. Further, the population is also sub-classified into tribes and castes based on cultural and social backgrounds [8]. These different layers of population stratification have led to the richness in diversity of India.

The genetic diversity is well reflected in the mitochondrial DNA (mtDNA), Y chromosomes, and candidate genes/markers, which have provided a fair understanding of the relatedness and divergence of specific communities or tribes of India [6, 8, 11–17]. The prevalence of consanguinity in marriages, due to cultural and social practices, in many sub-populations in India has led to the accumulation of genetic traits within communities [3, 18]. Studies have shown a high level of relatedness within subgroups suggesting accumulation of deleterious variations [19, 20]. These studies indicate that the ancestors of different subpopulations in India may have arisen from different waves of migration with relatively limited founding members, implying the source of genetic distinction, while regionally and culturally distinct groups continue to be genetically unique due to the practices of inbreeding.

A national genome-wide approach to understand the population architecture and look for markers specific to the Indian subcontinent was undertaken by the Indian Genome Variation (IGV) consortium, which used single-nucleotide polymorphisms (SNPs) to type 900 genes from over 1800 individuals across 55 endogamous populations. High heterozygosity values, varying allele frequencies, and common polymorphic haplotypes of sub-populations were shown to underline the heterogeneity within the Indian population. Additionally, unique mutations were discovered within the subcontinent, with concomitant founder effects [10, 21, 22].

The findings of the IGV consortium have led to the identification of specific markers and better understanding of genotype-phenotype correlations in Indian sub-populations. The phenotypically distinct outcomes of sub-population specific genotypes could be shown in susceptibility or resistance towards *Plasmodium falciparum* [23–27], risk of contracting glaucoma [28], homocysteine levels [29], and risk of developing high-altitude pulmonary edema [30, 31], among other examples. Further, case-control studies in ethnically matched groups as defined by IGV consortium allowed identification of Indian-specific susceptibility markers in genes causing Parkinson’s disease, Wilson disease, and albinism [32–35]. Sub-population-specific responses to various drugs have also been documented, based on differences in the allele frequencies of variants in metabolizer enzyme genes, across various ethnicities in India [36–38].

Thus, the extensive genetic heterogeneity and the endogamous cultural practices clearly suggest that there is a need to demarcate genetic affinities and distinctions among sub-populations. These findings also underscore the genetic distinction of the Indian population from the populations of other countries, warning against the imputation of genetic information from other populations. Evidently, a generalization of the population architecture can lead to erroneous interpretations in clinical settings.

### Genetic diversity of India: a driver of high-genetic disease prevalence

India, being a melting pot of genetic diversity, is also home to strict inbreeding practices and founder effects, which have resulted in the accumulation of deleterious genetic variations [39]. The reported prevalence of birth defects in India is 64.4 per 1000 live births [40]. The high genetic burden in India has been highlighted by independent studies [41–44]. The lack of a national newborn screening program until recently has led to a distending proportion of the Indian population ailing with genetic diseases [45]. Inborn errors of metabolism (IEM), which is a nation-wide issue, can be addressed on being identified at the neonatal stages [46, 47]. Hemoglobinopathies including sickle cell anemia, thalassemia, pose a significant burden in India, and are known in specific sub-populations [48, 49]. Down syndrome is another genetic disorder, which is the major cause of mental retardation, with a frequency of approximately 1 in 1000 births [50]. A database for cataloging genetic diseases, the Indian Genetic Disease Database (IGDD) has been set up, version 1.0 of which housed information on variants in 63 genes corresponding to 52 genetic diseases known in the Indian population [51]. The database is freely available and currently holds information on over 100 genetic diseases from around 3500 patients [52].

What is striking, apart from the high prevalence of monogenic diseases, is the heterogeneity in the outcome of the same disease. The clinical heterogeneity in blood disorders in India has been attributed to subpopulation-specific variations and allele frequencies [53–57]. Similarly, the phenotypic spectrum of Spinocerebellar ataxias (SCA) and their pathogenic variants have been shown across Indian subpopulations [42]. Ethnicity-dependent mitochondrial haplotypes have also been shown to give rise to differences in penetrance in the mitochondrial disease Leber’s hereditary optic neuropathy (LHON) [58]. Population-specific genetic variations and susceptibility to diseases have been shown in hereditary cardiomyopathy [59, 60] and drug/toxin metabolism [61]. The genetic heterogeneity, which was thought as an advantage, is, in fact, contributing to the high prevalence of genetic diseases in India. Several studies have also shown that the genetic variations and frequency information observed in population worldwide are not fully relevant to the Indian context [62–64]. Thus, it is important to document the true extent of genetic variation and burden of genetic diseases in Indian settings.

A number of genome-scale datasets of Indians have surfaced in recent years. These include an initiative by the IGV consortium of six laboratories affiliated to the Council of Scientific and Industrial Research (CSIR) with other key players, that typed SNPs and known markers scattered among 1000 genes [10, 21, 22, 65]. This was also followed by whole-genome sequencing of Indians from the USA [66] and from India [67, 68], in addition to several large-scale projects which sequenced healthy individuals who are descendants of Indian immigrants and from specific Indian sub-populations [69–72]. Genomes of healthy individuals from different parts of India were sequenced subsequently [73–77]. These initiatives have culminated in efforts to meta-analyze and integrate datasets, which has resulted in resources such as the South Asian Genomes and Exomes (SAGE) [76] and INDian EXome database (INDEX-db) [78]. In addition, several disease or application specific databases developed in India provide a rich source of information about the genetic diversity and underlying genetic disease prevalence in India (Table 1).

**Table 1:** Details of publicly available resources that can aid in rare genetic disease research in India

S. no.	Databases/resources	Description/URL	Reference
1	SAGE	A compendium of genetic variants integrating South Asian whole genomes and exomes http://clingen.igib.res.in/sage/	[76]
2	IGVdb	A DNA variation database of the people of India available to researchers for understanding human biology with respect to disease predisposition, adverse drug reaction, population migration, etc http://www.igvdb.res.in/index.php	[22]
3	MtBrowse	Integrative genomics browser for human mitochondrial DNA hosting genomic variation data from over 5000 individuals with 22 disease phenotypes http://ab-openlab.csir.res.in/cgi-bin/gb2/gbrowse	[79]
4	mit-o-matic	A comprehensive cloud-based tool for clinical evaluation of mitochondrial genomic variations from NGS datasets http://genome.igib.res.in/mitomatic/help.html	[80]
5.	INDEX-db	Database of genetic variations from the Indian population http://indexdb.ncbs.res.in/	[78]
6.	TMC SNPdb	First open source SNP database from whole exome data of 62 samples derived from cancer patients from India. http://www.actrec.gov.in/pi-webpages/AmitDutt/TMCSNP/TMCSNPdp.html	[81]
7.	IGDD	Indian Genetic Disease Database http://www.igdd.iicb.res.in/	[51]

It is to be noted that given the heterogeneity shown by IGV and other studies, the number of Indian genomes and exomes that are available till date under-represents the peninsula’s diversity. This gap in the availability of baseline genetic information can hence act as a barrier in understanding the causes of diseases that are prevalent in the country and calls for a nation-wide genome project, as being undertaken in other parts of the world [82].

## Main text

### Rare diseases: a significant burden for India

Rare diseases or orphan diseases are defined as those which afflict a minimal fraction of a population. An attempt to identify the parameters that can be used to define a rare disease was made by the ‘Rare Disease Terminology & Definitions Used in Outcomes Research Working Group.’ The study concluded that a disease with the average global prevalence of 40–50 cases per 100,000 people can be called as a rare disease [83]. The Orphan Drug Act (ODA) of 1983 [84] under the US law, which was instrumental in gathering attention towards rare diseases [85], defined a rare disease in the USA as a disease affecting fewer than 200,000 people of the total population. The council of the European Union defined a rare disease as 5 in 10,000 [86]. The rare disease prevalence for different countries thus varies. For instance, the respective rare disease prevalence numbers are 65 in 100,000 in Brazil [87], 1 in 2500 in Japan [83], and 33.2 per 100,000 in Taiwan [88].

The pervasive endogamy and founder effects in sub-populations have led to a high prevalence of autosomal recessive rare genetic diseases in India, compared to other parts of the world. While there is no appropriate standard definition to describe a rare disease in India, Indian Council of Medical Research (ICMR) has defined a disease as rare if it affects less than 1 person in 2500 individuals [89]. The Organization for Rare Diseases India (ORDI) has suggested a threshold of 1 in 5000 for defining rare diseases in India [90]. About 5000–8000 rare diseases have been documented all over the globe accounting for up to 6–8% of the global population [86]. Approximately, 40% of the rare diseases can be attributed to genetic factors [91]. These diseases together contribute to a significant number of individuals and the disease burden in a populous country such as India.

The estimation of the prevalence of rare genetic diseases across India is limited by the lack of a centralized clinical registry of patients with rare genetic diseases. However, extrapolating the numbers in the Indian scenario, the Foundation for Research on Rare Diseases and Disorders has estimated that about 70 million people are affected by rare diseases [92]. Rare diseases that have gained attention in the country include blood disorders, lysosomal storage diseases, primary immunodeficiency diseases, mitochondrial diseases, neurodegenerative diseases, and musculoskeletal diseases, among many others [89, 93]. A compilation of estimated prevalence/incidence of well-studied rare diseases in India has been included in Table 2.

**Table 2:** List of rare genetic diseases with estimated prevalence/ incidence in India

S. no.	Rare disease	Frequency in India	Measure of estimation	State/region	Reference	Global prevalence (Orphanet)
1	Hemophilia A	0.9 per 100,000	Prevalence	All across India	[94]	1–9/100,000 (ORPHA:98878)
2	Hemophilia B	0.1 per 100,000	Prevalence	All across India	[94]	1–9/100,000 (ORPHA:98879)
3	Sickle cell anemia	2–20%	Allele frequency	All across India	[57]	1–5/10,000 (ORPHA:232)
4	Beta thalassemia trait	3–4%	Carrier Prevalence	All across India	[95]	1–9/1,000,000 (ORPHA:848)
5	Parkinson's disease	6–53/100,000	Prevalence	All across India	[96]	Unknown (ORPHA:411602)
6	Duchenne muscular dystrophy and Spinal muscular atrophy	1 in 1400 male live births	Prevalence	Tamil Nadu, South India	[97]	NA
7	Cystic fibrosis	0.40%	Gene frequency	All across India	[98]	1–9/100,000 (ORPHA:586)
8	Epilepsy	2.5–11.9/1000	Prevalence	North, South, East India	[96]	–
9	Intellectual disability	10.5/1000	Prevalence	All across India	[99]	–
10	Skeletal dysplasia	19.6 per 10,000 newborns	Incidence	Karnataka, South India	[100]	< 1/1,000,000 (ORPHA:1858)

Note: The table provides a list of prevalent rare genetic disease studies carried out in India. While there were studies for many other diseases, they have been excluded since they do not represent the actual prevalence in the general population

Given the estimate of approximately 70 million people living with rare diseases, most of them undiagnosed, rare disease management contributes a huge burden for a developing country like India. The accurate socio-economic burden due to rare genetic diseases in India is unknown. Incidentally, the social impacts of hemophilia have been recorded adequately, in spite of an underestimated prevalence due to lower case reporting [94]. Other studies have shown that government interventions can reduce the out-of-pocket expenditure of patients [101, 102]. A recent study showed a yearly expenditure of transfusion-dependent thalassemics attending a tertiary care center in India, to be Rs. 41,514 to 1,51,800. This is equivalent to USD 629–2300 with an average of Rs. 74,948 (USD 1135), amounting to almost 40% of the annual income of an Indian family [103]. In recent years, several initiatives have been taken by Indian organizations, both government and non-government, to address rare diseases and the availability of orphan drugs to help ailing patients [104]. However, there are several challenges including physician training, availability of molecular diagnosis, standard treatment protocols, and availability of drugs, among others, that need to be addressed to reduce the rare disease burden in India.

### Population scale initiatives for addressing rare diseases in India

Despite over 70 million individuals being affected by rare diseases, India has limited resources committed to treating or understanding rare diseases. In recent years, Indian Council of Medical Research (ICMR) has taken a step towards bridging the gap between patients suffering from rare genetic diseases and healthcare providers by launching The Indian Rare Disease Registry. The registry acts as a common repository for data concerning rare disease patients throughout the country [105]. Furthermore, there are examples of how various organizations, both government and non-government, have developed programs for addressing the rare disease challenge in India. However, most of these efforts are towards specific diseases areas or are targeted to a certain sub-population. Some of the notable initiatives that cater to heterogeneous rare disease patients are highlighted in this section.

Molecular Diagnostics, Counselling, Care and Research Centre (MDCRC) is a not-for-profit charitable organization which takes a holistic approach to manage Duchenne Muscular Dystrophy (DMD) patients, mostly catering to individuals from the southern part of India (Tamil Nadu). MDCRC undertakes genetic counseling in addition to providing screening for DMD and Spinal Muscular Atrophy (SMA). A pilot study by MDCRC estimated the prevalence of DMD to be 2.4 times higher as compared to global estimates [97]. The Uttar Pradesh state government had taken the commendable initiative in the year 2009 by providing anti-hemophilic factors (AHF) free of cost at various centers in the state [106], while the Maharashtra state government has provided clotting factor concentrates (CFC) to the poor sections and emergency cases since 2012 [107]. According to the hemophilia federation of India, 69% of the country is covered by AHF support [108]. These have been successful initiatives for public health in specific rare disease settings. Institute of Medical Genetics and Genomics at the Sri Ganga Ram Hospital, Delhi provides a battery of tests for several rare diseases [109] including blood disorders, metabolic disorders, muscular dystrophies, and Down syndrome [110], among others.

Sanofi-Genzyme’s India Charitable Access Program (INCAP), Shire HGT's charitable access program in partnership with Direct Relief (a non-governmental organization), and Protalix Biotherapeutics have provided access to enzyme replacement therapy for lysosomal storage diseases in India [111]. Apart from these, there are a handful of commercial companies in India that offer genetic testing for rare genetic diseases, thus aiding the rare disease diagnosis requirements. In recent years, ORDI, a non-profit non-government organization in India, is providing a platform for individual rare diseases support groups to come together. They aim to set up patient registries and work with the government to create policies that are orphan disease centered. ORDI undertakes both Indian and global initiatives, and works together with at least 15 rare disease foundations/centers [90].

The Genomics for Understanding Rare Diseases: India Alliance Network (GUaRDIAN) at CSIR-Institute of Genomics and Integrative Biology (CSIR-IGIB), Delhi is a unique research initiative in India that uses the power of genomics to solve and understand rare diseases. Details about the GUaRDIAN program are elaborated in the next section. Apart from those listed above, several government research laboratories, hospitals, and not-for-profit organizations also provide specialized tests for a specific patient group or community (see Tables 3 and 4 for more details).

**Table 3:** List of major research centers working on rare diseases in India

S. no.	Research centers	Major rare disease research areas
1	All India Institute of Medical Sciences (AIIMS), New Delhi	A referral hospital with multiple specialties. Major rare disease areas include skin disorders, mitochondrial disorders, neurological disorders, cardiac disorders, developmental disorders, and pediatric disorders, among others
2	Amrita Institute of Medical Sciences and Research Centre, Cochin	Lysosomal storage disorders (LSDs), Werner syndrome
3	Anthropological Survey of India (ASI), Kolkata	Hemoglobinopathies
4	CSIR Central Drug Research Institute (CDRI), Lucknow	Progressive external ophthalmoplegia
5	CSIR Centre for Cellular and Molecular Biology (CCMB), Hyderabad	Mitochondrial disorders, hemoglobinopathies, infertility
6	Centre for Human Genetics (CHG), Bengaluru	Inherited metabolic diseases (IMDs), EB, lysosomal storage disorders
7	Christian Medical College and Hospital (CMC), Vellore	A referral hospital with multiple specialties. Major rare disease areas include blood disorders among others
8	Center for Genetic Studies and Research, MMM Hospital, Chennai	Rare chromosomal diseases
9	FRIGE’s Institute of Human Genetics, Ahmedabad	Hemoglobinopathies, musculopathies, neurodegenerative diseases, lysosomal storage disorders, among other genetic diseases
10	CSIR Indian Institute of Chemical Biology (IICB), Kolkata	Oculocutaneous albinism (OCA), Wilson disease (WD), autism
11	Indian Institute of Science (IISc), Bengaluru	Primary microcephaly, anencephaly, Parkinson’s disease, Wilson disease, and neuromuscular disorders
12	Indira Gandhi Institute of Child Health, Bangalore	Lysosomal storage disorders, Prader-Willi syndrome, and skeletal dysplasia, among other rare diseases
13	CSIR Institute of Genomics and Integrative Biology (IGIB), New Delhi	A specialized laboratory for research in rare genetic diseases including skin disorders, ataxias, cardiac disorders, neurological disorders, primary immunodeficiency disorders, endocrinology disorders, nephrological disorders, mitochondrial disorders, Wilson disease, hemoglobinopathies, lysosomal storage disorders, and developmental disorders, among others
14	Jawaharlal Institute of Postgraduate Medical Education and Research (JIPMER), Puducherry	A referral hospital with multiple specialties. Major rare disease areas include Werner syndrome, Fanconi anemia, split hand-split feet syndrome, skin disorders, incontinentia pigmenti
15	Jawaharlal Nehru University (JNU), New Delhi	GNE myopathy
16	JK Lone Hospital, SMS Medical College, Jaipur	A referral hospital with multiple specialties. Major rare disease areas include ectodermal dysplasias, skeletal dysplasias, neurological disorders, lysosomal storage disorders, and coagulation disorders, among others.
17	Kalawati Saran Children’s Hospital, New Delhi	Pediatric disorders
18	King Edward Memorial (KEM) Hospital, Mumbai	Hemoglobinopathies, LSDs, inborn errors of metabolism
19	LV Prasad Eye Institute, Hyderabad	Eye diseases
20	Manipal University, Manipal	A referral hospital with multiple specialties. Major rare disease areas include skeletal dysplasia, neurodegenerative diseases, metabolic disorders, bleeding disorders, and malformation syndromes, among others
21	Maulana Azad Medical College, New Delhi	LSDs, skeletal dysplasia, hemophilia, pediatric disorders
22	National Institute of Biomedical Genomics (NIBMG), Kalyani	Hemoglobinopathies, Wilson disease, DMD, eye disorders
23	National Institute of Mental Health and Neuro-Sciences (NIMHANS), Bangalore	Various rare neuromuscular disorders including limb girdle muscular dystrophy, amyotrophic lateral sclerosis, metabolic myopathies, rare congenital myasthenic syndromes, neuropsychiatric syndromes, SCA, mitochondrial disorders, and metabolic disorders
24	Nizam’s Institute of Medical Sciences, Hyderabad	A referral hospital for a variety of rare genetic disease such as LSDs, hemoglobinopathies, and neurodegenerative disorders, among others
25	Osmania University, Hyderabad	Rare chromosomal disorders
26	Post Graduate Institute of Medical Education and Research (PGIMER), Chandigarh	A tertiary referral hospital with multiple specialties. Major rare disease areas include immune diseases, bone diseases, and Wilson diseases, among others
27	Sankara Nethralaya, Chennai	Eye diseases
28	Safdarjung Hospital, New Delhi	Metabolic diseases, cardiac disorders
29	Sanjay Gandhi Postgraduate Institute of Medical Sciences (SGPGIMS), Lucknow	A referral hospital with multiple specialties. Major rare disease areas include LSDs, oro-facial-digital syndromes, neurodevelopment disorders, hemoglobinopathies, neurodegenerative disorders, and metabolic disorders, among others
30	Sir Ganga Ram Hospital, New Delhi	A referral hospital for LSDs, neurodegenerative disorders, IEMs, mitochondrial disorders, and other rare diseases.
31	Sri Chitra Tirunal Institute of Medical Sciences and Technologies, Thiruvananthapuram	A referral hospital for immune diseases, autoinflammatory diseases, LSDs, and cardiac diseases, among others
32	The Centre for DNA Fingerprinting and Diagnostics (CDFD), Hyderabad	Neuromuscular disorders, metabolic diseases, hemoglobinopathies, thrombotic disorders, triplet repeat disorders, and LSDs, among others
33	The Datta Meghe Institute of Medical Sciences (DMIMS), Wardha	Multiple rare diseases
34	All India Institute of Medical Sciences, Jodhpur	A referral hospital for several rare diseases including cystic fibrosis, and leucocyte adhesion defect, among others
35	University of Delhi South Campus, New Delhi	Inborn errors of metabolism, intellectual disability, and Parkinson’s disease

**Table 4:** A comprehensive list of rare disease organizations and resources that provide patient support [modified from [90]]

S. no.	Organizations	Websites
1	Alzheimers and Related Disorders Society Of India (ARDSI)	http://ardsi.org/
2	Birth Defects Registry of India	http://www.fcrf.org.in/bdri_abus.asp
3	Down Syndrome Federation India	http://downsyndrome.in/
4	Fragile X Society–India	http://www.fragilex.in/
5	Genetic Alliance	http://www.geneticalliance.org
6	Hemophilia Federation	http://www.hemophilia.in/
7	Indian Rett Syndrome Foundation	www.rettsyndrome.in
8	Indian Association of Muscular Dystrophy	www.iamd.in
9	Indian Prader-Willi Syndrome Association	https://ipwsa.in/
10	Indian Patients Society for Primary Immunodeficiency (IPSPI)	www.ipspiindia.org
11	Indian Organization for Rare Diseases (I-ORD)	http://www.i-ord.org/
12	Indian Society for Primary Immune Deficiency	http://www.ispid.org.in/
13	Lysosomal Storage Disorders Support Society (LSDSS)	www.lsdss.org
14	Metabolic Errors and Rare Diseases (MERD)	http://merdindia.com
15	Muscular Dystrophy Association India	http://mdindia.net/
16	Muscular Dystrophy Foundation India	http://www.mdfindia.org
17	Muskaan (intellectually disabled)	https://muskaanthengo.org/
18	National Thalassemia Welfare Society	http://www.thalassemiaindia.org/
19	Open Platform for Rare Diseases (OPFORD)	https://opford.org/
20	Organization of Rare Disorder India (ORDI)	https://ordindia.org/
21	Pompe Foundation	http://pompeindia.org/
22	Rare Diseases India	http://www.rarediseasesindia.org
23	Retina India	http://retinaindia.blogspot.com/
24	Sjogren’s India	http://www.sjogrensindia.org
25	Society for Hemophilia Care, India	http://www.shcindia.org/
26	Thalassemics India	www.thalassemicsindia.org

### GUaRDIAN

Completion of the human genome project and the availability of the human genome reference sequence have opened up opportunities for a new era of genomic medicine. This has a tremendous impact on diagnosis, treatment, and preventive care related to genetic diseases [112–114]. The decade after the completion of the human genome sequence has ushered in significant technological advancements [115–117]. These technologies, popularly known as Next Generation Sequencing (NGS) technologies have enabled fast sequencing of genomes at an affordable cost [118, 119]. The improvements in technology have also contributed immensely to the development of complementary methods towards extraction of biological interactions between biomolecules including the transcriptome [120–122] and epigenome [123]. In addition, the integration of personal omics data provides opportunities to view the temporal dynamics of omics profiles in an individual [124, 125]. These advances have brought in a paradigm shift in current practices of medicine. Genome sequencing has significantly impacted the understanding of genetic variants and their association with diseases. Recently, exome and genome sequencing are increasingly being used to investigate the genetic bases of diseases including both monogenic as well as complex diseases such as cancer. One of the major applications of such genomic technologies in the clinical setting is the identification and annotation of variants associated with rare genetic diseases [126–130]. A rare disease patient usually undergoes three misdiagnoses and takes up to 7 years to reach the right diagnosis [131]. With genome sequencing technologies, it is now possible to look at either the entire genome or the protein-coding regions (exomes) that may harbor deleterious variations, in a reasonable time. Given the presence of unique variations in Indian populations, absent elsewhere in the world, genomics-based solutions are the way forward to tackle the high burden of rare diseases. Identifying the causative variant(s) in rare genetic diseases would be important not only in enabling accurate diagnosis but also in counseling and genetic screening applications.

**Fig. 1:**
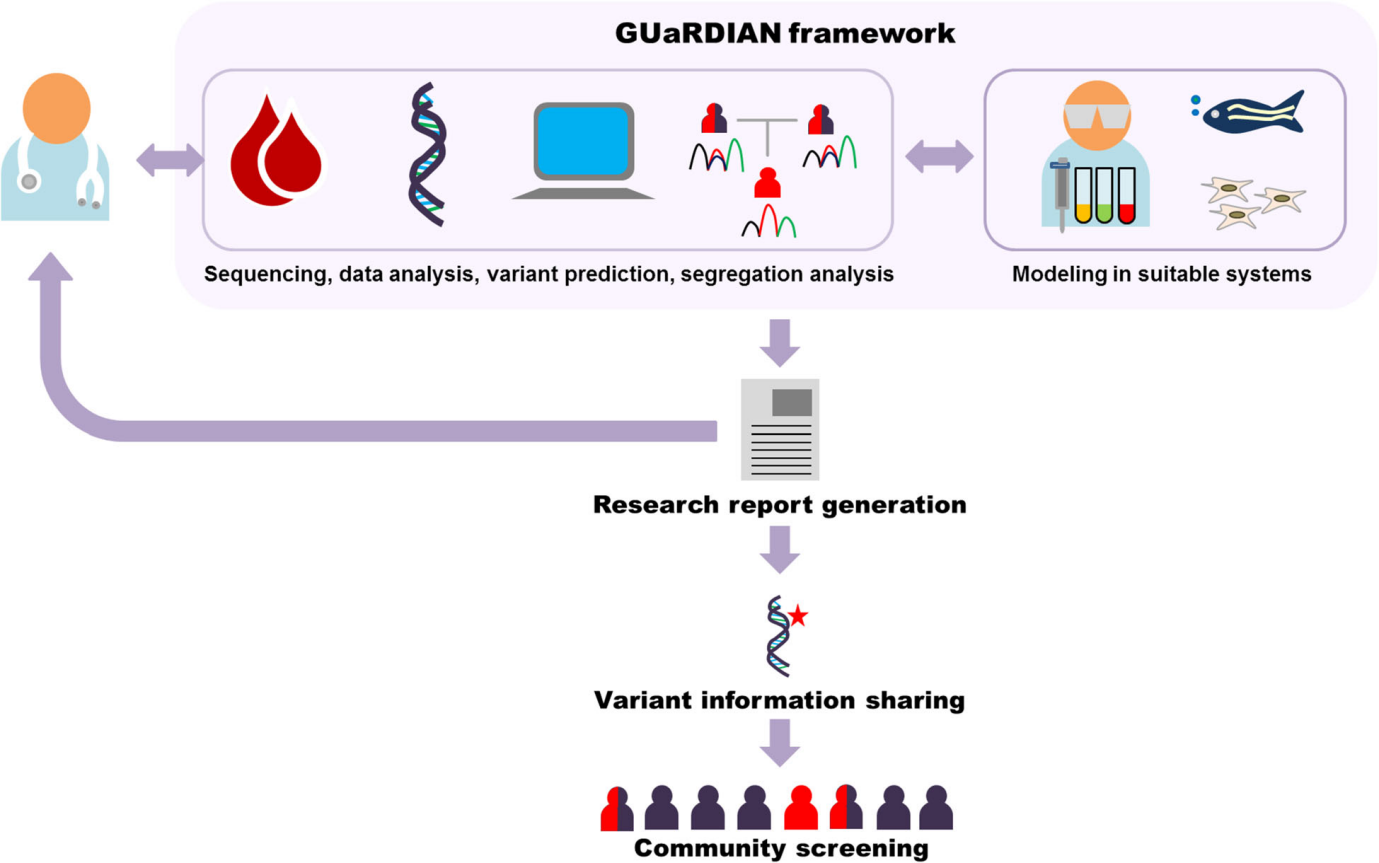
The GUaRDIAN framework. Clinicians refer patients and family members to GUaRDIAN consortium following which the blood/DNA samples and complete clinical investigations are shared. The samples undergo next generation sequencing, bioinformatic analyses, and variant prediction. The predicted genetic variant is checked for segregation in the family members using capillary sequencing. If a known pathogenic variant is identified, a research report is generated and sent back to the clinician. When a putative novel variant is identified, the effect of the genetic variant is modeled in a suitable system to validate the functionality of the variant and also to understand the disease mechanism. Further, the genetic variant information derived from patient/family is made available for community-level screening

The major challenges in realizing the full potential of genomics technologies for identifying genetic disease-causing variants in India are manifold. These include the uniqueness of the Indian genetic pool, lack of a program for identifying rare genetic diseases, and a comprehensive registry of rare genetic diseases, logistics of sample procurement and processing, common protocols for genome sequencing and computational analysis, and methodologies for validating the functionality of the reported variation(s). Genomics for Understanding Rare Diseases: India Alliance Network (GUaRDIAN) is a research consortium which was proposed to address the above challenges. The consortium includes clinicians, clinical geneticists, genomics scientists, computational analysts, and basic research biologists, among others. The clinicians and clinical geneticists form the primary contacts and act as caregivers for the patients. The geneticists, genomics scientists, and researchers provide the necessary expertise required to identify the genetic variations, create models for understanding disease mechanisms, and explore the therapeutic potential of small molecules for rare genetic diseases. The simplified workflow of the GUaRDIAN consortium is summarized in Fig. 1. The GUaRDIAN is an open-ended consortium of individuals, who are actively invited to join the consortium, with an agreement to follow the general principles and framework, and the data access policies. A common framework for the exchange of datasets, resources within the consortium, and participatory approach has been proposed to realize the full potential of clinical genomics.

The aim of the GUaRDIAN consortium is to establish a unique collaborative framework in health care planning, implementation, and delivery in the specific area of rare genetic diseases. The consortium proposes to apply the power of genomics for systematic characterization and diagnosis of rare genetic diseases in India. The GUaRDIAN network is connected to hospitals and major tertiary care centers across India. The consortium currently encompasses over 240 clinicians/researchers, from 70 clinical/research centers across India [132]. The GUaRDIAN is a research program and not a clinical service.

#### GUaRDIAN ethical framework

A strong foundation of an ethical and legal framework is necessary for seamless collaboration and sharing of genetic data across the boundaries of institutions. The GUaRDIAN consortium is strongly anchored on the basic principles of beneficence, reciprocity, justice, and professional responsibility. As part of the collaborators’ network, a common format for collection of clinical and genetic data has been created. Additional efforts have gone into standardizing the patient information. The benefits and potential ethical, legal, and social implications of whole exome or genome sequencing and availability of the anonymized data in the public domain are conveyed in detail to the patients and family. The identity stripped clinically annotated data of variations is available to all the members through a firewalled access. In addition, publications in peer-reviewed journals serve as the major interaction points for sharing findings with the general clinical and research community.

#### GUaRDIAN clinical registry

As part of the collaborative initiative, a referral system for systematic collection and curation of baseline data is being maintained. The program collects detailed clinical information, including the signs, symptoms, and clinical investigations performed on the patient and family members. The GUaRDIAN maintains a semantically oriented framework, which relies extensively on the internationally accepted and popularly used semantic ontologies established and widely used including the human phenotype ontology [133]. The application of such a centralized data resource is manifold. While on the one end, it not only provides a holistic view of the burden of genetic diseases in the country, it also provides immense insights into the common and rare genetic variants in different sub-populations. This would enable clinicians and policy-makers to design intervention programs including genetic education and genetic counseling.

#### GUaRDIAN sequence data generation

A centralized sequencing facility has been established at the CSIR-Institute of Genomics and Integrative Biology (CSIR-IGIB), Delhi, which can be accessed by any collaborator in order to generate high-quality NGS sequencing data as per international standards [134–136], with various platforms such as Hiseq 2500 and NovaSeq 6000 (Illumina Inc. USA). A dedicated training team for both experimental and computational work necessary to perform the data capture and analysis of high-throughput sequencing data is also channelized as a part of the GUaRDIAN consortium. Investigators are free to generate sequence data on their own or from other commercial facilities that adhere to international guidelines and GUaRDIAN consortium standards. The sequencing requirements are updated and modified in accordance with the technological advancement and emerging international consensus.

#### GUaRDIAN data analysis, integration, interpretation, and sharing

GUaRDIAN stands for providing scientifically sound and clinically actionable solutions. The genomes/exomes of patients are analyzed through custom built in-house bioinformatic pipelines to identify the most accurate genetic variation that can explain a certain condition. Further, the pathogenicity of variants is predicted by the latest guidelines laid down by the American College of Medical Genetics and Genomics [136]. The GUaRDIAN consortium relies heavily on datasets, tools, and resources developed across the whole world, including methods and tools developed as part of the OpenPGx consortium [137, 138]. The consortium depends on open source architectures, tools, and open access resources, to enable easy replication, scalability, and future implementation in independent clinical setups.

Data sharing also forms a major component of the program and collaboration. The anonymized clinically annotated data of variations is available to all members through a firewalled access. In addition, the summary data of each novel variant and/or allele frequencies would be available in the public domain without access restrictions. Credits for contributions are a major point to address in such a scalable collaborative network. All collaborating members of the network shall agree to adhere to basic principles of data veracity and ethical codes of conduct. The credit-sharing agreement forms the major framework of trust between participating members. This shall be in line with principles laid out for biomedical resource contributions [139].

#### GUaRDIAN reporting, community screening, and disease modeling

Once the GUaRDIAN computational analysis identifies a pathogenic variation of clinical significance, it is subjected to validation by segregation analysis. After this, if the identified genetic variation is immediately actionable, the information is transferred to the clinician as a research report which will be used for patient counseling. This genetic information can further be used for making informed decisions by the family. Wherever required, the genetic variation information is utilized for potential community-level screening programs, thus building towards affordable diagnostic solutions.

In the case where novel pathogenic variations are identified, researchers at the GUaRDIAN consortium replicate the disease in suitable models such as zebrafish and patient-derived IPSCs to gain the correlation between the disease phenotype and the identified variant. Genetic engineering to create disease models also provides the opportunity for discovery of novel therapeutics as well as to repurpose existing drugs for new indications in rare genetic diseases.

#### GUaRDIAN success stories

A large number of cases have been solved through the GUaRDIAN program, and a subset of interesting investigations have been published in peer-reviewed journals, which encompass diseases as diverse as epidermolysis bullosa [140–143], familial Mediterranean fever [144], lamellar ichthyosis [145], sporadic acrokeratosis verruciformis [146], rare syndromes of mineralocorticoid excess [147], severe combined immunodeficiency [148], X-linked agammaglobulinemia [149], hyper IgE syndrome [150], Dowling-Degos disease [151], and megalencephalic leukoencephalopathy [152], to list a few. Furthermore, GUaRDIAN is actively investigating the genetic conundrum in Indian rare disease cohorts conforming to cardiology, neurology, dermatology, primary immunodeficiency, endocrinology, nephrology, mitochondrial disorders, and lysosomal storage disorders, among others.

Of the many success stories of GUaRDIAN, the diagnosis of a rare mutation in* megalencephalic leukoencephalopathy with subcortical cysts 1 (MLC1) *gene in leukodystrophy was instrumental in community service in the form of affordable diagnostics. Six children from a consanguineous Muslim family belonging to the Nalband community from north India were presented with difficulty in balancing the head and inability to sit independently, with recurrent episodes of seizures. Based on the clinical characteristics, the provisional diagnosis of leukodystrophy was made; however, leukodystrophies are a class of disorders with the involvement of multiple genes. Whole exome sequencing revealed a homozygous variation in the *MLC1* gene, found to be segregated among all the affected members and was absent in all the unaffected members. Based on this, the diagnosis of megalencephalic leukoencephalopathy with subcortical cysts (MLC) was confirmed. MLC is a rare leukodystrophy characterized by macrocephaly, progressive motor dysfunction, recurrent episodes of seizures, and mental retardation. Further, three more families from the same community were found to be affected and carried the same variation, indicating a founder effect. As a follow up for this, an additional 83 members of the community were screened. Out of these, 24 were found to be the carriers and 9 were affected [152]. The Nalband community consists of over 5000 members scattered across north India as well as Pakistan. Like many other communities in India, consanguineous marriages are common in the Nalband community. In order to aid the entire community, a polymerase chain reaction (PCR)-based assay for the Nalband mutation in *MLC1* has been developed for carrier status determination and prenatal screening, at an affordable cost.

Another area where the GUaRDIAN has made a significant contribution is in the rare diseases of the skin. Epidermolysis bullosa (EB), a skin-blistering disease, was once considered ultra-rare in the Indian population. Epidermolysis bullosa simplex (EBS) is the most common subtype of EB. The GUaRDIAN team identified a novel variant in the *Keratin 5 (KRT5)* gene in a large multigenerational family from northwestern India. The variant was shown to be segregated in nine affected members in the family but found absent in five unaffected members. The study reported the first causative mutation for EBS from India [140]. Whole exome sequencing has also enabled the detection of a novel homozygous nonsense variant in *Keratin 14 (KRT14)* gene in an autosomal recessive form of EB, in two siblings presented with generalized blistering of the skin and dystrophic nails. The same study identified a known homozygous stop gain variant in the same gene in a child with trauma-induced blistering all over the body [153]. In cases of junctional epidermolysis bullosa (JEB) and dystrophic epidermolysis bullosa (DEB), the phenotype and genotype spectrum of the disease was described for the first time from India through collaborative efforts of GUaRDIAN. JEB was studied in a small cohort of six patients from four consanguineous families with a wide range of clinical variability, identifying variations in the genes *laminin subunit alpha 3 (LAMA3)*, *laminin subunit β3 (LAMB3)*, *collagen type XVII α1 (COL17A1)* [142]. In the case of DEB, 18 patients from 17 unrelated families were studied and 20 distinct variations were found in *COL7A1* gene [143]. There have also been other reports which discovered novel variants that expanded the known mutation spectrum of EB [141, 154].

GUaRDIAN has contributed to the identification of the pharmacogenetic variants in *dihydropyrimidine dehydrogenase (DPYD)* gene, which determines the metabolism of the commonly used anti-neoplastic drug 5-fluorouracil, in south-east Asian countries [155]. The consortium has also undertaken international initiatives to derive the pharmacogenomic landscape in Malays [156] and Qatari populations [157, 158], and to identify genetic variants of Arab, Middle East, and North African populations [159, 160]. GUaRDIAN has also set up a systematic pipeline for next generation sequencing of the mitochondrial genome for clinical applications, called the mit-o-matic [80].

In the era of clinical genomics, it is imperative for clinicians to be well equipped with the basics of high-throughput data analysis so as to interpret the data concerning a certain disease. Keeping this in mind, the GUaRDIAN consortium initiated an outreach program, where clinicians are trained in basics of NGS technologies and systematic computational analysis of sequencing data as a part of continuing medical education (CME) workshops. A handbook called ‘Exome Sequence Analysis and Interpretation for Clinicians’ has been prepared and made available for free download from Google Books [161]. Over 8000 soft copies of the book have been downloaded and over 800 print copies have been distributed to clinicians in meetings and CMEs (as of January 2019). More than 500 clinicians have been trained across the country. The GUaRDIAN outreach program is a small step towards providing health and economic benefits to families with rare genetic diseases.

### Impact of genomics in diagnosis of rare genetic diseases in India

It has been increasingly shown that the challenges of genetic and phenotypic heterogeneity which makes diagnosis of rare genetic diseases cumbersome could potentially be addressed by using next generation sequencing techniques, enabling the high-throughput identification and annotation of causal variants [126, 129, 162, 163]. In the present scenario, the rare diseases which require immediate attention in India are primary immunodeficiencies, hemoglobinopathies, muscular dystrophies, metabolic disorders, and neurological disorders, among others. The earlier section described the contributions made by a genomics-enabled nation-wide network, GUaRDIAN. There have also been other individual genomics-based studies that have aided in addressing rare diseases.

In the case of Duchenne muscular dystrophy (DMD), a wide spectrum of mutations and frequencies have been shown in patients from different Indian sub-populations [164–166]. The dystrophin gene spans over 2000 kb at the DNA level, with pathogenic variations identified within introns as well. Traditional methods based on multiplex ligation-dependent probe amplification (MLPA) have been used to detect carrier status in DMD [167–170]. A recent study showed that NGS can be used in the diagnosis of muscular dystrophies in MLPA negative cases with a success rate of as high as 100% [171].

Lysosomal storage disorders (LSD), a class of more than 50 genetic diseases, are found to be of high burden in India [172]. The overlapping phenotypes and involvement of multiple genes in lysosomal disorders, and the need for intervention in the form of enzyme replacement therapy at the earliest, call for use of NGS approaches for faster diagnosis. In Niemann–Pick disease type C, an LSD with a wide clinical spectrum, a novel mutation was identified by whole exome sequencing in a proband of Asian origin, which was a deletion spanning two exons of *Niemann–Pick disease type C2 (NPC2)* gene [173].

An estimated one million Indians are affected by primary immunodeficiencies, a class comprising of hundreds of genetic disorders [174]. The utmost challenging facet of PIDs is under diagnosis, owing to the high incidence of infectious diseases in countries like India [175]. Whole exome sequencing approach has proved to be instrumental in identifying mutations in capillary sequencing negative cases of X-linked agammaglobulinemia (XLA) [149], severe combined immunodeficiency (SCID) [148], B cell expansion with NF-κB, and T cell anergy (BENTA) [176], apart from targeted next generation sequencing in SCID [177] and major histocompatibility complex class II deficiency [178].

Mitochondrial disorders are difficult to diagnose owing to overlapping phenotypes and multi-system involvement. Whole mitochondrial genome sequencing coupled with nuclear gene sequencing has been performed to establish genotype-phenotype correlations in a cohort of patients from South India [179]. Whole exome sequencing has incidentally helped in diagnosing mitochondrial diseases due to nuclear genome variations [180, 181].

In case of autosomal recessive forms of ataxia, such as spastic ataxia [182] and cerebellar ataxias [183], homozygosity mapping as well as whole exome sequencing has played a major role in discovering the novel variants in Indian patients. Application of genomic diagnosis has been appreciated for skeletal dysplasias in a recent study. The study on a large cohort using capillary sequencing as well as NGS has added novel variants to the existing literature [184]. Exome sequencing also has been used to discover novel mutations in multiple joint dislocation syndrome [185], Schwartz-Jampel syndrome type 1 [186], and progressive pseudorheumatoid dysplasia [187]. Currently, a limited number of clinicians are using NGS-based diagnosis of rare genetic diseases in India but this number is increasing at a rapid pace. With several success stories emerging from India, genomics will become a mainstay for diagnosis of rare genetic diseases in the near future.

### Translating genomics to affordable diagnostics for rare genetic diseases

Although the cost of next generation sequencing-based diagnostics is declining, with more than 70 million people suffering from a genetic disease in India, affordable and faster measures are required to cater to the needs of the ailing population. CSIR-IGIB has an ongoing outreach platform to provide affordable access to genetic testing for common genetic diseases. The program named “Genomics and other Omics tools for Enabling Medical Decision (GOMED)” [188] provides molecular genetic assays for clinical diagnosis, prenatal testing, and carrier screening. In this ‘from bench to bedside’ model, a battery of low-cost genetic diagnostic assays for diseases pertaining to neurology, cardiology, and many other disorders are available. Till now, over 90 candidate gene tests and 7 comprehensive gene panel tests have been developed by GOMED. Over 20,000 molecular tests for about 6000 patients have been performed across the country (As of 2018). This clinical service is provided free of cost to needy patients. GOMED has been particularly beneficial in the community screening of sub-population-specific mutations. Whole exome sequencing had revealed a founder mutation in *MLC1* gene in individuals from Nalband community suffering from megalencephalic leukoencephalopathy with subcortical cysts (MLC) [152]. As part of GOMED, a low-cost diagnostic assay was developed to screen for carriers in other members of this community comprising of 5000 people scattered across different regions in north India. Spinocerebellar ataxia (SCA) type 3, known as Machado–Joseph disease (MJD) is one of the most common ataxias globally, while presenting rarely in India. Intervention by CSIR-IGIB revealed the hidden burden of SCA3/MJD in 100–200 families in a close-knit community in Maharashtra. This information is now available as an assay under GOMED. GOMED also expands to pharmacogenetic testing to prevent adverse reactions to commonly used drugs such as the anticancer drug 5-fluorouracil. 5-fluorouracil (5-FU) is an anti-neoplastic drug which is administered in a number of cancers, the clearance of which is mediated by a rate-limiting enzyme dihydropyrimidine dehydrogenase (DPYD). Genotyping of four variants in* DPYD* gene that were found to be associated with 5-FU toxicity in South Asian population [155] has been made available as an affordable diagnostic assay for testing cancer patients before administering the drug to prevent adverse reactions. The GOMED program also actively works with commercial diagnostic companies to provide technologies for the affordable diagnosis of common and rare genetic diseases in India.

As a step towards improving public health, efforts have also been undertaken to compile a directory of genetic test services and counseling centers in India. The directory includes about 120 centers across various states in India. It acts as a resource for clinicians as well as researchers for referring to facilities which provide accessible and comprehensive public healthcare [189].

### The way ahead

There are a few priority areas that are emerging in the country as far as rare diseases are concerned. Newborn screening at a nation-wide level is pivotal in reducing the burden of rare diseases. In 2014, India Newborn Action Plan (INAP) was released to reduce the incidence of child birth defects and stillbirths [190]. While at present, there are limitations in implementing genomics-based diagnosis at population scale [191], Indian pediatricians are hopeful about the genomic interventions and resultant advancements in diagnosis, especially for non-invasive prenatal testing [192]. National Policy for Treatment of Rare Diseases was released by the Indian Ministry of Health and Family Welfare in 2017 [193]. However, this policy was withdrawn in November 2018 to the utter dismay of the patients and family members suffering from rare diseases [194]. As personal genome-sequencing becomes popular, it is important to create a policy and a legal framework for non-discrimination of individuals based on the genetic information. This would be in line with the Genetic Information Nondiscrimination Act (GINA) of the USA but also adapted to the social and cultural sensibilities specific to India. As we look ahead, we should involve stakeholders such as government policy-makers, research scientists, clinicians, hospitals, patient groups, and non-governmental organizations to join forces to find meaningful solutions for rare diseases patients.

For a large and heterogeneous population like that of India, it has been shown that the international genomics initiatives such as the 1000 genome project have an inadequate representation of the genetic diversity due to limited sampling [20]. In highly endogamous populations such as the Ashkenazi Jewish population, genomics has been crucial in understanding rare diseases with founder effects [195]. With an enormous and stratified population, practicing extensive endogamy [39], it is expected that India would have a high prevalence of rare genetic diseases. Therefore, it is essential to know the causal genes and pathogenic genetic variants and the sub-populations where they are prevalent, to aid in the appropriate and cost-effective diagnosis of rare diseases. There are several initiatives in India that are attempting to address this space by building large-scale whole genome datasets of the representative population. Programs such as the GenomeAsia100K, which has representative samples from India, seek to sequence and analyze individuals to help enable medical applications [196]. The Government of India has announced a Bioscience Mission for Precision Health and Optimal Well-being, which will involve large-scale human genome sequencing across India [197]. Towards this, the Council of Scientific and Industrial Research (CSIR), India, has also initiated a whole genome sequencing program titled “Genomics for Public Health (IndiGen)” [198] to help accelerate biomedical applications in India. These population scale genomics programs will definitely provide the momentum and ecosystem for driving rare disease genomics in India.

## Conclusion

India is home to culturally and genetically diverse populations, which are burdened by genetic diseases. Due to the high prevalence of recessive alleles owing to endogamous practices, rare diseases form a significant burden in India. Genomics can greatly aid in addressing rare disease burden by faster and more accurate diagnoses. The Genomics for Understanding Rare Diseases: India Alliance Network (GUaRDIAN) provides a template for a nation-wide collaborative platform that uses the power of genomics to dissect the rare disease conundrum. More such pan-India genomics-driven initiatives can help in deriving Indian-specific references for deducing pathogenic and benign variations in the population, which can pave the way for precision medicine, including in the rare disease space.

## Data Availability

Data sharing is not applicable to this article as no datasets were generated or analyzed during the current study.

## Abbreviations

5-FU: 5-Fluorouracil
AHF: Anti-hemophilic factors
BENTA: B cell expansion with NF-κB and T cell anergy
CFC: Clotting factor concentrates
CME: Continuing medical education
COL17A1: Collagen type XVII α1
CSIR: Council of Scientific and Industrial Research
DEB: Dystrophic Epidermolysis Bullosa
DMD: Duchenne Muscular Dystrophy
DPYD: Dihydropyrimidine dehydrogenase
EB: Epidermolysis bullosa
EBS: Epidermolysis bullosa simplex
GOMED: Genomics and other Omics tools for Enabling Medical Decision
GUaRDIAN: Genomics for Understanding Rare Diseases: India Alliance Network
ICMR: Indian Council of Medical Research
IGDD: Indian Genetic Disease Database
IGIB: Institute of Genomics and Integrative Biology
IGV: Indian Genome Variation
INAP: India Newborn Action Plan
INCAP: India Charitable Access Program
INDEX-db: INDian EXome database
JEB: Junctional Epidermolysis Bullosa
KRT: Keratin
LAMA3: Laminin subunit α3
LAMB3: Laminin subunit β3
LSD: Lysosomal storage disorders
MDCRC: Molecular Diagnostics, Counseling, Care and Research Centre
MJD: Machado Joseph Disease
MLC: Megalencephalic Leukoencephalopathy with subcortical Cysts
MLPA: Multiplex ligation-dependent probe amplification
NGS: Next generation sequencing
NPC2: Niemann-Pick disease type C2
ORDI: Organization for Rare Diseases India
PCR: Polymerase chain reaction
SAGE: South Asian Genomes and Exomes
SCA: Spinocerebellar ataxia
SCID: Severe combined immunodeficiency
SMA: Spinal muscular atrophy
SNP: Single nucleotide polymorphism
XLA: X-linked agammaglobulinemia
